# Rabies-preventive health program for mothers in maternal and child health centers: quasi experimental study

**DOI:** 10.1186/s41043-025-00740-6

**Published:** 2025-01-17

**Authors:** Samia Ibrahim Mabrouk Baraka, Rania Abd Elmohsen Abo El Nour, M. A. Abdelzaher, Eman Mahmoud Seif El–Nasr

**Affiliations:** 1 Community Health Nursing Department, Faculty of Nursing, Tanta University, Tanta, 31527 Egypt; 2 Community Health Nursing Department, Beni-Suef Health Technical Institute, Ministry of Health, Beni-Suef, 62511 Egypt; 3 Anesthesia Techniques Department, College of Health and Medical Techniques, Al-Mustaqbal University, 51001 Babylon, Iraq; 4 Environmental Science and Industrial Development Department, Faculty of Postgraduate Studies for Advanced Sciences, Beni-Suef University, Beni-Suef, 62511 Egypt; 5 Community Health Nursing Department, Faculty of Nursing, Cairo University, Cairo, 11562 Egypt

**Keywords:** Rabies prevention, Quasi-experimental study, Mothers’ awareness

## Abstract

**Background:**

Rabies spreads to people and animals via saliva, usually through bites, scratches, or direct contact with mucosa (e.g. eyes, mouth, or open wounds). Rabies remains a major public health problem in Egypt, with an estimated 60 people dying annually from the disease.

**Aim:**

The aim of this study was to assess the effect of preventive program on mothers' awareness for prevention of rabies.

**Study design:**

Quasi-experimental research design was utilized in this study.

**Setting:**

This study was conducted at three major Maternal and child centers (Botors, Embaby and Segar) in Tanta City.

**Sample:**

A total sample of 150 mothers who attended the child clinic with children aged 6–12 years.

**Tools:**

Researchers developed three tools for data collection. The tools underwent content validity assessment, and their internal consistency was evaluated using Cronbach's alpha coefficient. Structured interviewing questionnaire for assessment of mothers’ knowledge, attitudes and practices regarding rabies **Tool I**, Mothers' knowledge regarding rabies questionnaire. This tool consisted of two parts: demographic characteristics of the studied mothers and mothers’ knowledge about rabies. **Tool II**, Rabies attitudinal Likert scale. **Tool III,** Mothers reported practice Questionnaire.

**Results:**

From the obtained results, it was found that, a statistically significant correlation was found between mothers' total knowledge scores, attitude and total practice scores, both before and after the implementation of the rabies prevention program.

**Conclusion:**

It was concluded that the rabies- preventive health program had a positive impact on mother’s knowledge, attitude and practices regarding rabies. The mean knowledge score improved from 14.08 before preventive program to 20.90 one month posttest. Negative attitude of the studied mothers was 48% before program but it reduced to 1.3% one month after preventive program. Only 13.35% of the participants had satisfactory level of practice before program and increased to 83.30% one month posttest.

**Recommendations:**

Dissemination of the program through mass media campaigns and community outreach activities to enhance public knowledge, improve attitudes, and promote better practices regarding rabies prevention and control measures.

## Introduction

Rabies is a neglected viral zoonotic disease that affects people in many parts of the world, causing a significant mortality burden, especially in poor countries. It is responsible for approximately 60,000 deaths each year. More than 150 countries across all continents, except Antarctica, have reported cases of rabies. The disease burden is predominantly reported in Asia and Africa, with the majority of cases occurring after dog bites, particularly in rural areas. Children under 15 years of age account for approximately 40% of cases. This viral infection has a substantial impact on public health, especially in developing nations where access to proper medical care and preventive measures may be limited [14].

Rabies is an acute, preventable viral disease of the nervous system in mammals. It is caused by a rhabdovirus (species Rabies virus of the genus Lyssavirus) and is usually transmitted through the bite of a rabid animal already infected with it. The disease is typically characterized by increased salivation, abnormal behavior, paralysis, and death when not treated promptly [9, 24]. Rabies infects mammals, including dogs, cats, livestock, and wildlife. The virus spreads to people and animals via saliva, usually through bites, scratches, or direct contact with mucous membranes (e.g., eyes, mouth) or open wounds. It is a deadly virus that causes progressive and fatal inflammation of the brain and spinal cord. Once clinical symptoms appear, rabies is virtually 100% fatal. Vaccinating dogs is the most cost-effective strategy for preventing rabies in people [6].

All cases of dog bites are considered suspected cases of rabies, which can be confirmed through clinical observation and/or laboratory testing. The clinical confirmation of rabies is based on a history of dog bite followed by classical symptoms such as anxiety, agitation, paralysis, excessive salivation, and hydrophobia. The incubation period for rabies is typically 2–3 months but may vary from 1 week to 1 year. This period depends on factors such as the exposure site (how far it is from the brain), the type of rabies virus, viral load, and any existing immunity. The initial symptoms of rabies may be very similar to those of flu, including general weakness or discomfort, fever, and headache [2].

The Sustainable Development Goals (SDGs) of the 2030 agenda include actions targeting improvements in health and education and ending epidemics of neglected tropical diseases (NTDs) in the next decade. To support these goals, it is crucial to develop tools to increase rabies control through effective use of vaccines in animals, medicines, tools, and technologies, while generating innovations and measuring impact [11]. Egypt has established a thorough strategic plan to eradicate human rabies transmitted by dogs by 2030. The strategy encompasses several key components which includes enhanced multi-sectoral approach nationwide, animal rabies elimination through dog population management, mass dog vaccination programs, improved post-exposure prophylaxis management, strengthened surveillance systems, community awareness and education and resource mobilization [7, 8].

Community health nurses play a vital role in rabies prevention through multiple key functions that directly impact program success. The nurses' work directly contributes to program outcomes by increasing community awareness about rabies prevention measures, ensuring proper and timely administration of post-exposure treatment, which is nearly 100% effective in preventing human rabies deaths when properly administered. So, community health nurses serve as critical links between healthcare systems and communities, helping to achieve the global goal of eliminating human deaths from rabies through prevention, education, and proper medical intervention [5].

Rabies prevention programs often involve community health nurses working to educate the public about rabies risks and prevention strategies. These nurses may conduct awareness campaigns, provide information on proper animal handling and vaccination, and coordinate with local veterinary services to promote dog vaccination efforts [13].

Mothers play an important role in their children's lives, particularly in disease prevention. They are crucial in ensuring access to health care for their children and families. Increasing their awareness about rabies and preventive measures can help prevent the occurrence of the disease. However, mothers may have insufficient knowledge about rabies prevention and management. Lack of understanding regarding rabies complications, dangers, wound management, and vaccination has been a significant factor in rabies-related deaths among children [17]. Community health nurses in maternal and child health centers play an important role in helping mothers reduce mortality and morbidity of their children. Health education to mothers and families about rabies prevention and protection by rabies vaccination to house animals as a primary level of prevention. Early detection of rabies cases and early treatment and applying the appropriate first aid to the wound as a second level of prevention. Isolation of rabies cases in dark and quiet environment as a tertiary level of prevention [1].

## Significance of the study

Rabies remains a major public health problem in Egypt, with an estimated 60 people dying annually from the disease. There are over 200,000 animal bites recorded each year in the country, mostly from dogs. It disproportionately affects vulnerable populations, particularly children aged 5–14 years, who represent 40% of people bitten by suspected rabid animals [5]. The disparity between scientific understanding and practical implementation has created several critical gaps in rabies control efforts. Translating this knowledge into effective practice across different settings remains problematic. In many regions, there are significant behavioral and structural barriers that limit the effectiveness of preventive strategies. These include low awareness of rabies severity, misconceptions about vaccines, and inadequate access to healthcare resources. A major challenge lies in the multi-sectoral nature of rabies control, requiring coordination between animal welfare, public health, veterinary medicine, and civil administration sectors [20]. Limited research has been conducted on rabies and its prevention among mothers in maternal and child health centers in Egypt. Many Observations indicate that these mothers may generally lack knowledge, proper practices, and appropriate attitudes regarding rabies. Consequently, the researcher aims to highlight rabies prevention [21] So, the aim of this study was to assess the effect of preventive program on mothers' awareness for prevention of rabies. Operational definition: Mothers' awareness of rabies was assessed through their knowledge, attitudes, and practices.

## Materials and methods

### Research hypotheses

- *H.1* Mean knowledge score of mothers after implementation of the rabies- preventive health program, will be higher than before.
- *H.2* Mean attitude score of mothers after implementation of the rabies- preventive health program, will be higher than before.
- *H.3* Mean practice score of mothers after implementation of the rabies- preventive health program, will be higher than before.

### Research design

A quasi-experimental one-group pretest–posttest design was utilized in the current study. This type of quasi-experimental design was used by researchers when only one group is available for study. In this design, data are collected at two time points: once before implementing the program (pretest) and again one month after implementation (posttest) on the same group of subjects [23].

### Setting of study

The study was conducted at three major Maternal and Child Health Centers (Botros, Embaby, and Sager) in Tanta City. These centers were chosen because they are the largest maternal and child health facilities in the area and have a high frequency of mothers and children attending them.

### Sample

A purposive sample of 150 mothers who attended the child clinic with their children aged 6–12 years in the previously mentioned settings. The sample size was computed using Raosoft sample size calculation soft program, where, N is the total mothers who attended the three maternal and child health centers with the inclusive criteria within three months of data collection process, 90% confidence level, 5% margin of error. A power of 0.90 minimizes the likelihood of a Type II error, providing a 90% probability of detecting a statistically significant effect if one exists. The flow rate of eligible mothers at the selected settings was 300 within three months. While the calculated sample size was 143, it was increased to 150 to account for potential dropouts. The final sample comprised 50 mothers from each of the three maternal and child health centers to enhance the study’s internal validity. To minimize selection bias, participants were recruited from multiple health centers, and baseline characteristics were compared to ensure group equivalency. Use different statistical methods to control confounding variable sampling. Inclusion criteria: All mothers attended to child clinic in previously mentioned maternal and child health centers with their children aged 6–12 years and agree to participate in the study.

### Tools of data collection

Three tools were utilized in the current study, developed through an extensive review of literature. A structured interview questionnaire used to assess mothers' knowledge, attitudes, and practices regarding rabies.

#### Tool (I): mothers knowledge regarding rabies questionnaire

This tool consisted of three parts:

##### 1st part

1. Socio-demographic characteristics: included mothers age, marital status, education, occupation, place of residence and income.
2. Source of information regarding rabies disease
3. Mothers information about pets and bites: dog ownership, vaccination of the dog, reasons for un-vaccination, family history of doges' bite, actions done with the bitten person and actions done with the animal that bitted person.

##### 2nd part

This part was developed by the researcher after reviewing current literature [4], to assess mothers' knowledge of rabies. The assessment covered various aspects of the disease, including its definition, modes of transmission, animals capable of transmitting rabies, signs and symptoms in both animals and humans, anti-rabies vaccine dosage, vaccine effectiveness, and administration routes. Responses to these questions were evaluated one month after implementing the preventive program through a post-test.

**Scoring system**: Scoring system was designed to assess mothers' knowledge, is calculated as follow:

- One point awarded for each correct answer and zero points for incorrect or "don't know" responses. The total possible score ranged from 0 to 26 points with greater points indicate good level of knowledge.

##### 3rd part, The total score of knowledge

It was determined by taking points as the following: -

**Table:** 

Total score categories	Percent (from total score)	Points
Low level	< 60	0–15
Moderate level	60- < 75	16–19
Good level	≥ 75	20–26

#### Tool II: rabies attitude likert scale

This part was developed by the researcher after reviewing current literature, including Taha et al. [18] to assess mothers' attitudes towards rabies. It consisted of 12 items with a scoring system as follows: 2 points for "agree," 1 point for "slightly agree," and 0 points for "disagree." The total scores were calculated by summing up individual item scores and converting them to percentages. The total attitude score ranged from 0 to 24, with higher scores indicating more positive attitudes. The total score was formulated as the following:

**Table:** 

Total score cateogories	Percent (from total score)	Points
Negative attitude	< 60	0–13
Neutral attitude	60%—< 75	14–17
Positive attitude	≥ 75	18–24

#### Tool III: mothers reported practice questionnaire

It was developed by researchers based on recent literature, particularly the works of Teferi [19] and Taha et al. [18]. It aimed to evaluate mothers' reported practices regarding animal bites and rabies. The tool covered several key areas, including immediate first aids measures following a bite, proper wound care techniques, the importance of seeking medical attention, appropriate responses when dealing with potentially rabid individuals or animals, and strategies for rabies prevention and control.

##### Scoring system

The scoring system for reported practices was calculated as follows: a score of (1) was assigned for completed practices, while a score of (0) was given for practices not performed. The scores for individual items were summed and then divided by the total number of items to obtain a mean score. These mean scores were subsequently converted to percentages. The maximum possible score for practice was 18 points. The total practices score is classified according to the following.

**Table:** 

Total practices score	Percent	Points
Unsatisfactory level	< 75	0–13
Satisfactory level	≥ 75	14–18

### Ethical consideration

Official permission was obtained from the directors of three Maternal and Child Health Centers (Botros, Embaby, and Sager; Code# 477-5-2024). Mothers were informed about the study's purpose and significance. The researchers emphasized that participation was entirely voluntary. Anonymity and confidentiality were assured, and oral informed consent was obtained from the mothers. All participants were advised that they could withdraw at any time without penalty. Ethical committee approval was secured from the Faculty of Nursing.

### Phases of application of the Program: there were four phases

1. *Assessment phase* During this stage, researchers conducted a pre-test assessment of mothers. A structured interview questionnaire used to assess mothers' knowledge, attitudes, and practices concerning rabies at the previously specified Maternal and Child Health centers. Data collection took place four months from May to Septamber 2024. The researchers introduced themselves and used simple Arabic language when administering the questionnaire. They were present at the centers two days per week (Saturdays and Tuesdays) on a rotating schedule, from 9 AM to 12 PM. In each visit, data was gathered from around 5–7 mothers per day for half an hour for each mother.
2. *Planning Phase* This phase included the arrangement for conduction of the program such as setting objectives, creating the content of the program including the session's goals, teaching place, sessions, teaching methods and handouts. Then, the program and the educational materials were prepared based on related literature reviews about rabies. The researchers used simple teaching methods such as power point presentation containing videos and pictures to present the rabies preventive program contents. A colorful booklet was given to every mother.
3. *Implementation Phase* In this phase, the rabies-preventive health program was implemented based on pre-test assessment data. The preventive health program was conducted for one month (June 2024); through which the researchers met mothers three days per week (Saturdays, Tuesdays and Thursday) from 9 AM to 12 PM to complete the program content. Mothers who shared in the program divided into groups; each group consisted of 10–15 mothers in each session. Three sessions were given to mothers in the form of teaching classes and the expected duration of each session was from 30 to 45 min. Motivation, open discussion and reinforcement were used during the session to enhance learning. At the end of each session, open-end questions were asked to ensure that there was no misunderstanding occurred and for more explanation for unclear information. Summary was done after each session and at the end of the program. Then a booklet containing the main points was distributed to the students at the end of the program.

*The first and the second sessions* At the start of the first session, the researchers welcomed the mothers and thanked them for participating in the study. They inquired about the mothers' expectations regarding the program and then provided an overview of the total number of sessions. These sessions covered theoretical aspects of rabies, including its definition, the animals that transmit the disease, signs and symptoms, prognosis, and information about the anti-rabies vaccine.

*The third session* It covered several aspects of rabies management and prevention. It outlined proper first aid procedures following an animal bite, including wound care techniques. The importance of seeking medical attention was emphasized. Also, the session included appropriate behaviors when encountering potentially rabid individuals or animals and various control measures for preventing the spread of rabies.

(4) Evaluation phase The evaluation was conducted using a post-test administered one month after the completion of the preventive rabies program. This evaluation aimed to measure the mothers' knowledge, attitudes, and practices regarding rabies prevention.

### Validity and reliability

The content validity of the tools was evaluated by a panel of five expertise professors from the Faculty of Nursing at Tanta University, specializing in community health nursing. The researchers submitted the study tools to these experts to examine all items and assess their relevance to the study's hypotheses and objectives. Each expert reviewed the tools for content coverage, clarity, wording, length, format, and overall appearance. Modifications were made based on the panel's feedback. The content validity ratio was calculated through this formula: CVR= (N_e_ - N/2)/(N/2), in which the N_e_ is the number of panelists indicating "essential" and N is the total number of panelists. The numeric value of content validity ratio is determined by Lawshe Table. In this study, the number of panelists were 5 members,‏ so the value of CVR were 0.99 which considered good and perfect content . To assess reliability, Cronbach's alpha test was used to measure the internal consistency of the tools, resulting in a Cronbach's alpha coefficient of 0.897.

### Pilot study

Pilot study conducted on (10%) of the sample to ensure the clarity and applicability of the content of tools. This pilot sample was included in the study as there were no modifications made to the tool.

### Statistical analysis

The collected data underwent analysis and tabulation using the Statistical Package for Social Sciences (SPSS) version 23. Results were presented in figures, utilizing number and percentage distributions, means, and standard deviations. Appropriate statistical tests were employed to determine the presence of significant relationships. Chi-square (χ^2^) tests were used for qualitative data analysis, while Pearson correlation coefficients (r) were calculated for correlation analysis. The effect size (Cohen’s d) for a paired-samples t-test can be calculated by dividing the mean difference by the standard deviation of the difference. The degree of significance was identified using p-values, with the following interpretation: highly significant (*p* < 0.001), significant (*p* < 0.05), and not significant (*p* > 0.05).

## Results

The study results will be presented in the following sequence:

1. Part I: Description of demographic characteristics of mothers.
2. Part II: Distribution of mothers according to their knowledge, attitude and practice about rabies in pre and post-test.
3. Part III: Correlation between study variables in pre and post-tests.

Table 1 represents that mothers' demographic characteristics revealed a diverse age range from 20 to 59 years, with a mean age of 38.56 ± 9.55. Slightly more than half (56.7%) of the mothers were under 40 years old, while 14% were over 50 years old. Regarding marital status, the majority (86.7%) were married, with 9.3% being divorced. Educational attainment varied, with approximately half (55.3%) having completed secondary education, in contrast to only 3.3% holding postgraduate degrees. The data also highlighted that a significant proportion of the studied mothers were unemployed (60.7%), resided in rural areas (64.7%), and reported insufficient income (62.7%).

**Table 1:** Distribution of the studied mothers according to their demographic characteristics

Demographic characteristics	No (150)	%
1. Mothers' Age (in years)		
< 40	85	56.7
40–< 50	44	29.3
≥ 50	21	14
2. Range: 20–59 Mean ± SD: 38.56 ± 9.55		
3. Marital Status		
Married	130	86.7
Widow	6	4
Divorced	14	9.3
4. Educational		
Illiterate	35	23.3
Basic education	0	0.0
Secondary	83	55.3
University	27	18.0
Postgraduate	5	3.3
5. Occupation		
Working	59	39.3
Not Working	91	60.7
6. Residence		
Urban	53	35.3
Rural	97	64.7
7. Mothers Income		
Not adequate	94	62.7
Adequate	38	25.3
Adequate and save	18	12

**Fig. 1:**
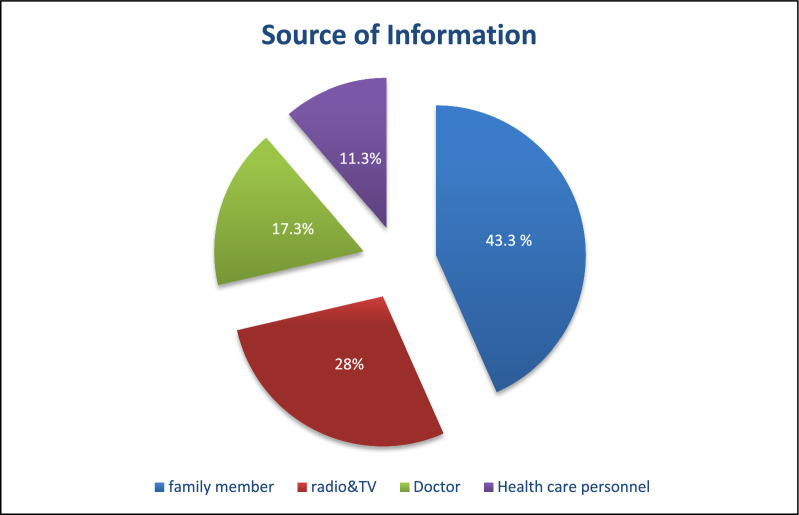
Percentage distribution of the studied mothers according to source of information regarding rabies disease

Figure 1 shows that 43.3% of the studied mothers reported that their source of information was from family members, followed by radio and television (28%), heath care personnel (11.3%) was the least contribution as source of information.

Table 2 represents that less than two thirds (65.3%) did not own animals compared with 34.7% owned animals at their homes. Among pet-owning mothers, the distribution was evenly split between dog and cat ownership. Concerning vaccination of the dogs, the table shows that 73.1% of the studied mothers who had dogs, didn't vaccinate their dogs, as 28.9% of them reported that vaccination is expensive and the dogs were healthy, no attention and there was no nearly veterinarian clinic were other causes of no dogs vaccination by about one fifth of them (21.1%). 39.3% of the studied mothers had family history of animals’ bites who reported that clean the bitten wound and contact a veterinarian were the first actions for bitten person by 100% followed by contact a traditional healer (98.3%), then going to hospital by 38.9%. As regards actions were done with the dog, 69.5% of the studied mothers from who had family history of animal bites, reported that they did not do anything for the dogs who rabid the human being.

**Table 2:** Distribution of the mothers according to information about pets and bites

	N (150)	%
1. Owning a pet at home		
Yes	52	34.7
No	98	65.3
2. Type of pet at home (n = 52)		
Dogs	26	50
Cats	26	50
3. Vaccination of pet (n = 52)		
Yes	14	26.9
No	38	73.1
4. Reasons of unvaccinated (n = 38)		
No attention	8	21.1
No nearby veterinarian clinic	8	21.1
It is expensive	11	28.9
The animal is healthy	11	28.9
There is no awareness about vaccination of dogs	0	0.0
5. Family history of animal bites		
Yes	59	39.3
No	91	60.7
6. Actions were done with the bitten person (n = 59)^(*)^		
Vaccination/a shot	22	37.3
Go to the hospital	23	38.9
Clean the bite wound	59	100
Contact a traditional healer	58	98.3
Report it to the dog owner	16	27.11
Contact a veterinarian	59	100
Kill the dog	5	8.47
Nothing	0	0.0
7. Actions were done with the dog: (n = 59)		
Animal under quarantine/died under observation	8	13.6
Killed by community	10	16.9
Nothing	41	69.5

(*) More than one answer was allowed

Table 3 demonstrates that 65.3% of the studied mothers had low level of knowledge regarding rabies disease, compared to 12% of mothers who had high level of knowledge before program. After program implementation within one month 76.7% of the studied mothers had a high level of knowledge compared to 6.7% of them had low level. There was statistically significant difference between before and one month later as Cohen’s d = 2.214; *p* < 0.001).

**Table 3:** Distribution of the studied mothers according to their total knowledge scores regarding rabies disease (N 150)

Mothers’ total knowledge Scores	Before		One month posttest		Effect size (Cohen's d)	P-value
	No	%	No	%		
Low (< 60%)	98	65.3	10	6.7	2.214	< 0.001*
Moderate (60%: < 75%)	34	22.7	25	16.6		
High (≥ 75%)	18	12	115	76.7		
Range Mean & SD	3–26 14.08 ± 4.80		11–26 20.90 ± 3.12			

*Significant at *p* < 0.05

Table 4 indicates that slightly less than half (48%) of the studied mothers had negative attitude regarding rabies disease, compared with 31.3% of them had positive attitude before program. After the program with one month there was 88% of the studied mothers had positive attitude, 10.7% of them had neutral attitude compared with 1.3% had negative attitude. There was statistically significant improvement in the total mean of attitudinal score before and one month later as the total mean and SD was 13.74 ± 4.64 before program and it changed to be 22.60 ± 3.24 one month later with Cohen’s d = 2.272; *p* < 0.001).

**Table 4:** Distribution of the studied mothers according to their total attitudinal scores regarding rabies disease (N 150)

Mothers’ total attitudinal Scores	Before		One month posttest		Effect size (Cohen's d)	P-value
	No	%	No	%		
Negative attitude (< 60%)	72	48	2	1.3	2.272	< 0.001*
Neutral attitude (60–< 75%)	31	20.7	16	10.7		
Positive attitude (≥ 75%)	47	31.3	132	88		
Range Mean & SD	0–24 13.74 ± 4.64		13–24 22.60 ± 3.24			

*Significant at *p* < 0.05

**Fig. 2:**
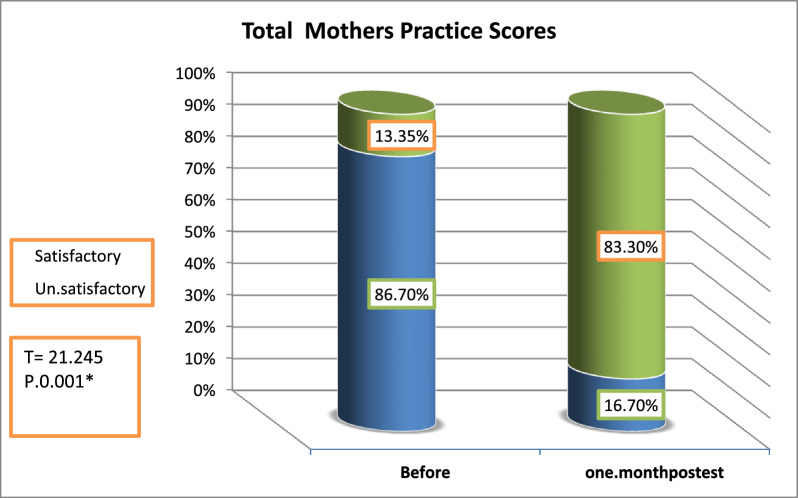
percentage distribution of the studied mothers regarding total practice score regarding rabies disease

Figure 2 shows that the majority (86.70%) of the studied mothers had unsatisfactory level of practice regarding rabies disease, compared to 13.35% of them had satisfactory level of practice before program. After program with one month, there was 83.30% of the studied mothers had satisfactory level of practice compared to 16.70% of them had unsatisfactory level. There was statistically significant difference between before and one month later as *p* = 0.001.

Table 5 demonstrates that there was statistically significant difference between total knowledge score, mothers' age, occupation, residence and income before and one month later preventive program (*p* < 0.05). There was statistically significant difference between total knowledge score, marital status and education before preventive program application only (*p* < 0.05).

**Table 5:** Relation between total knowledge scores regarding rabies disease and scio demographic characteristics among mothers in maternal and child health center (N 150)

Scio-demographic characteristics	Before			X^2^\ P	One month posttest			X^2^\ P
	Total Knowledge Score				Total Knowledge Score			
	Low (n = 98)	Moderate (n = 34)	High (n = 18)		Low (n = 10)	Moderate (n = 25)	High (n = 115)	
	No (%)	No (%)	No (%)		No (%)	No (%)	No (%)	
Age								
< 40	55 (56.1)	20 (58.8)	10 (55.6)	17.869 0.001*	5 (50)	5 (20)	75 (65.2)	22.846 0.001*
40– < 50	29 (29.6)	14 (41.2)	1 (5.6)		5 (50)	11 (44)	28 (24.3)	
50 and more	14 (14.3)	0 (0.0)	7 (38.9)		0 (0.0)	9 (36)	10 (10.4)	
Marital status								
Married	96 (98)	17 (50)	17 (94.4)	52.478 0.001*	9 (90)	25 (100)	96 (83.5)	5.308 0.258
Widow	0 (0.0)	6 (17.6)	0 (0.0)		0 (0.0)	0 (0.0)	6 (5.2)	
Divorced	2 (2)	11 (32.4)	1 5.6)		1 (10)	0 (0.0)	13 (11.3)	
Education								
Illiterate	33 (33.7)	2 (5.9)	0 (0.0)	99.50 0.001*	3 (30)	9 (36)	23 (20)	7.406 0. .285
Secondary	64 (65.3)	16 (47.1)	3 (16.7)		5 (50)	15 (60)	63 (54.8)	
University	1 (1)	11 (32.4)	15 (83.3)		2 (20)	1 (4.)	24 (20.9)	
Postgraduate	0 (0.0)	5 (14.7)	0 (0.0)		0 (0.0)	0 (0.0)	5 (4.3)	
Occupation								
Working	49 (50)	0 (0.0)	10 (55.6)	28.701 0.001*	1 (10)	18 (72)	40 (34.8)	15.788 0.001*
Not Working	49 (50)	34 (100)	8 (44.4)		9 (90)	7 (28)	75 (65.2)	
Residence								
Urban	50 (51.)	0 (0.0)	3 (16.7)	31.819 0.001*	1 (10)	14 (56)	38 (33)	7.749 0.023*
Rural	48 (49)	34 (100)	15 (83.3)		9 (90)	11 (44)	77 (67)	
Mothers’ income								
Not adequate	52 (53.1)	34 (100)	8 (44.4)	26.719 0.001*	9 (90)	6 (24)	79 (68.7)	43.819 0.001*
Adequate	31 (31.6)	0 (0.0)	7 (38.9)		1 (10)	19 (76)	18 (15.7)	
Adequate and save	15 (15.3)	0 (0.0)	3 (16.7)		0 (0.0)	0 (0.0)	18 (15.7)	

X^2^ Chi square test

^*^Significant at *p* < 0.05

Table 6 illustrates that there was statistically significant difference between total practice score, education, occupation and residence before and one month later preventive program (*p* < 0.05). there was statistically significant difference between total practice score, mothers' age and income one month test only (*p* < 0.05), where 52.8% & 55.2% of mothers who had satisfactory level of practice, their age were less than 40 years old and did not have adequate income respectively. On the contrary, there was no statistically significant difference between total practice score and marital status before and one month after of program implementation (P > 0.05).

**Table 6:** Relation between total practice score regarding rabies disease and sociodemographic characteristics among mothers in maternal and child health center (N 150)

Socio-demographic characteristics	Before		X^2^\ P	One month posttest		X^2^\ P
	Total Practice Score			Total Practice Score		
	Un satisfactory (n = 130)	Satisfactory (n = 20)		Unsatisfactory (n = 25)	Satisfactory (n = 125)	
	No (%)	No (%)		No (%)	No (%)	
Age						
< 40	69 (53.1)	16 (80)	5.422 0.067	19 (76)	66 (52.8)	6.471 0.039*
40– < 50	42 (32.3)	2 (10)		6 (24)	38 (30.4)	
50 and more	19 (14.6)	2 (10)		0 (0.0)	21 (16.8)	
Marital Status						
Married	110 (84.6)	20 (100)	3.553 0.169	20 (80)	110 (88)	1.582 0.453
Widow	6 (4.6)	0 (0.0)		1 (4)	5 (4)	
Divorced	14 (10.8)	0 (0.0)		4 (16)	10 (8)	
Education						
Illiterate	35 (26.9)	0 (0.0)	11.988 0.007*	2 (8)	33 (26.4)	36.373 0.001*
Secondary	65 (50.)	18 (90)		8 (32.)	75 (60)	
University	25 (19.2)	2 (10)		15 (60)	12 (9.6)	
Postgraduate	5 (3.8)	0 (0.0)		0 (0.0)	5 (4)	
Occupation						
Working	56 (43.1)	3 (15)	5.725 0.017*	0 ((0.0)	59 (47.2)	19.452 0.001*
Not Working	74 (56.9)	17 (85)		25 (100)	66 (52.8)	
Residence						
Urban	38 (29.2)	15 (75)	15.899 0.001*	3 (12)	50 (40)	7.147 0.008*
Rural	92 (70)	5 (25)		22 (88)	75 (60)	
Mothers’ income						
Not adequate	77 (59.2)	17 (85)	5.579 0.061	25 (100)	69 (55.2)	17.879 0.001*
Adequate	35 (26.9)	3 (15)		0 (0.0)	38 (30.4)	
Adequate and save	18 (13.8)	0 (0.0)		0 (0.0)	18 (14.4)	

X^2^ Chi square test

*Significant at *p* < 0.05

Table 7 presents a statistically significant correlation between total knowledge scores and total practice scores before and also one month posttest as it was (r = 0.231, *p* = 0.005) before program implementation, while in one month posttest was (r = 0.222, *p* = 0.006). Concerning relation between total knowledge score and total attitude scores before program, there was no statistically significant correlation between total knowledge scores and total attitude scores ( r = 0.066, *p* = 0.424), Although in one month after of the program, showed a statistically significant correlation (r = 0.259, *p* = 0.001), on the other side, there was no statistically significant correlation between total attitude scores and total practice scores before and one month posttest (r = 0.125, *p* = 0.129 & r = 0.134, *p* = 0.102) respectively.

**Table 7:** Correlation between total mothers, knowledge scores, total attitude scores and reported practice scores (N 150)

Time	Total Knowledge Score		Total practice score	
	R	p	R	p
Before				
Total practice score	0.231**	0.005	–	–
Total attitude score	0.066	0.424	0.125	0.129
One Month After				
Total practice score	0.222**	0.006	–	–
Total attitude score	0.259**	0.001	0.134	0.102

**Correlation is highly significant at the 0.01 level (2-tailed)

*Correlation is significant at the 0.05 level (2-tailed)

## Discussion

The World Health Organization's latest strategic plan for achieving the Sustainable Development Goals focuses on eliminating neglected tropical diseases between 2021 and 2030. A key target in this roadmap is to have 92% of countries achieve zero human rabies deaths by 2030. Rabies, classified as a neglected tropical disease, has a significant impact, particularly in rural regions. It affects over 150 countries worldwide, including Egypt, and is responsible for an estimated 59,000 human fatalities each year. This zoonotic disease continues to be a major public health concern, especially in underserved areas [22].

Rabies is a serious zoonotic disease that is almost always fatal once clinical symptoms appear. However, it is preventable through effective interventions, including awareness and proper management practices in communities. The implementation of educational programs to improve knowledge, attitudes, and practices (KAP) regarding rabies prevention and control is crucial, especially for mothers who play a key role in protecting their children's health. So, the current study aimed to evaluate the effect of a preventive program on mothers who attended child clinic in maternal and child center in Tanta City.

Based on the search results, the socio-demographic characteristics of the participating mothers revealed several issues. More than half of the mothers were under 40 years of age, indicating a relatively young sample population. This age distribution may have implications for the mothers' knowledge, attitudes, and practices regarding rabies and its prevention. Regarding educational level, over half of the mothers had completed secondary education. This educational level might be attributed to the fact that two-thirds of the participants resided in rural areas, where access to higher education is often limited. The prevalence of secondary education among the mothers could influence their understanding of health-related issues, including rabies.

These findings aligned with a study conducted by Elkholy et al. [5], which assessed mothers' knowledge and attitudes concerning rabies and its preventive measures. Their research similarly found that approximately two-fifths of the mothers had attained secondary education. This consistency in educational levels across studies suggests a potential pattern in the educational background of mothers in similar geographical or socioeconomic contexts. However, it is important to note that these results differ from those reported by Bihon et al. [3] in their study of rabies knowledge, attitudes, and practices in and around South Gondar, Northwest Ethiopia. Their research indicated that only 15% of their sample had completed secondary education, a significantly lower proportion than observed in the current study. This discrepancy highlights the potential variability in educational attainment across different regions and emphasizes the importance of considering geographical regions when interpreting and comparing such data.

The current study found that over three-fifths of mothers were not working and had inadequate income. The high proportion of non-working mothers with inadequate income suggests potential barriers to accessing healthcare services and preventive measures. These findings were partially supported by Elkholy et al. [5], who reported similar results regarding employment status but differed on income adequacy. This high rate of unemployment among mothers may be attributed to various factors such as cultural norms, limited job opportunities, or personal choices related to childcare responsibilities.

Regarding the main source of information about rabies, over two-fifths of mothers obtain information from family members. This may result from limited access to various common information sources for rural residents. The high reliance on family members for rabies information can be attributed to several key factors affecting rural information access. Rural areas face significant infrastructure limitations, including inadequate electricity, telecommunications, roads, and transportation systems, which restrict access to diverse information sources. These findings were inconsistent with those of Khalaf and Khalaf [13], in their study assessing the effect of a health education program on rabies knowledge for households in rural areas of Assiut Governorate, Egypt. Their research revealed that more than two-fifths of the sample had acquired knowledge about rabies from radio and television. This discrepancy in information sources may result from family members playing a significant role in information sharing in some areas but mass media channels like radio and television appear to be more influential in others.

The present research revealed that above one-third of mothers owned pet animals, with around three-quarters of them not vaccinating their animals. Above a quarter of mothers reported that vaccination is expensive, and animals are healthy. Regarding actions taken with dogs after a bite, above two-thirds of mothers reported that nothing was done with the dogs. These findings underscore the urgent need for community education about pet vaccination importance, affordable veterinary care access, and proper post-bite management protocols. In contrast with these results, Hagos et al. [12], in their study assessing knowledge, attitude, and practice towards rabies and associated factors among household heads in Mekelle city, Ethiopia, revealed that above three-quarters of household heads had vaccinated their dogs. The disparity in vaccination rates between the two studies suggests a potential difference in knowledge and attitudes towards rabies prevention. A higher level of awareness and positive attitudes towards rabies prevention among household heads.

The study revealed that the percentage of mothers with a high level of knowledge increased significantly after program implementation, rising from a minority to over three-quarters of participants (*p* = 0.0001). The significant improvement in knowledge scores may be attributed to the interactive nature of the sessions which led to mothers retaining and understanding rabies information more effectively. This finding aligned with research conducted by Sudarnika et al. [15] on "The Success of the 'Kasira' Rabies Cadres in Improving Community Knowledge and Attitudes towards Rabies," which reported an increase in the percentage of participants with good rabies knowledge from over one-quarter to about three-quarters (26.5% to 72.5%). Similarly, a study done by Khalaf and Khalaf [13] found that while most households (94.5%) had poor knowledge scores in the pretest, after the program implementation, two-thirds (66.1%) achieved good scores. The significant improvement observed highlights the potential impact of well-designed program on mothers’ health awareness and knowledge.

Regarding mothers' total attitude scores concerning rabies, the study results showed that approximately half of the mothers initially had a negative attitude toward the disease. However, after the program, the majority reported a positive attitude about rabies. Additionally, a statistically significant difference was found between the mean attitudinal scores before and one month after the program. The shift from negative to positive attitudes is particularly notable because attitude changes often require both knowledge acquisition and addressing underlying fears or misconceptions about the disease. This change may be attributed to most mothers realizing that rabies is a dangerous and serious disease. The study conducted by Elkholy et al. [5], found that 93.1% of the studied mothers had poor knowledge scores regarding rabies. Additionally, 57.5% of the mothers had indifferent total attitude scores concerning rabies. This indicates that a majority of mothers initially lacked adequate knowledge and had neutral attitudes towards the disease.

In this study, the majority of mothers initially reported unsatisfactory practices about rabies. However, one month after the program, their practice levels had improved significantly and became satisfactory. A statistically significant difference was found between the results before the program and one month after its implementation. The significant improvement in practice levels one month post-intervention demonstrates the value of structured educational programs in enhancing rabies prevention behaviors. Elkholy et al. [5] found that 76.7% of mothers initially had unsatisfactory practices regarding animal bites and rabies. After an educational program, there were "statistically significant relations between mothers' total knowledge, total practice and total attitude" related to rabies.

According to relation between the studied group knowledge & their socio-demographic characteristic, the present study revealed a highly statistically significant difference in total knowledge scores in relation to mothers' age, occupation, and income before and one month after the preventive program. These findings were supported by Sivagurunathan et al. [16] in their study on " Knowledge, attitude, and practice study on animal bite, rabies, and its prevention in an urban community" in India reported that,there were highly statistically significant relations between demographic characteristics of the studied mothers and their age, occupation and family income reported highly statistically significant relationships between demographic characteristics of the studied participants and their age, occupation, and family income. These findings highlight the importance of considering socio-demographic factors when designing and implementing preventive programs for rabies awareness and prevention. Also, a statistically significant correlation was found between mothers' total knowledge scores, attitude and total practice scores, both before and after the implementation of the rabies prevention program. This finding suggests that increased knowledge about rabies is associated with improved preventive practices. These results emphasize the importance of rabies education programs that not only enhance knowledge but also focus on transforming improved attitudes into preventive actions.

Regarding practice scores, significant differences were found between total practice scores and education level, occupation, and place of residence both before and one month after implementation of the rabies prevention program. These findings emphasize that socioeconomic factors like education, occupation, and residence significantly influence how people implement rabies prevention measures. Additionally, statistically significant differences emerged between total practice scores and mothers' age and income after the program. Conversely, there was no statistically significant difference between total practice scores and marital status either before or one month after program implementation. The association between education level and practice scores is consistent with previous research. A study by Hagos et al. [12] reported that educational level was a significant factor influencing knowledge and practices related to rabies. The finding of significant variations in practice scores between urban and rural areas aligns with previous studies, Gebrezgiher et al. [10] reported significant differences in rabies knowledge and practices between urban and rural populations. This highlights the importance of considering geographical factors when designing rabies prevention programs.

The study faces two key limitations as potential response bias from self-reporting methods, and insufficient data on long-term knowledge retention and behavioral changes due to the absence of extended monitoring. Rural areas with high rabies occurrence could benefit from this program's replication, given its uncomplicated structure and economical deployment requirements Upcoming studies need to examine how effectively the program sustains rabies prevention practices over time and determine whether incorporating it into maternal and child healthcare centers is viable. So, community education programs show promise in decreasing rabies cases, and their nationwide expansion could support WHO's 2030 rabies elimination goals, particularly in countries like Egypt where rabies remains prevalent.

## Recommendations

Based on the finding of the current study, the following recommendations are suggested:

- Introducing rabies’ knowledge, correct practices and first aid measures in different community settings.
- Dissemination of the program to the public through mass media to increase their knowledge; improve attitude and practices regarding rabies and its preventive measures.
- Raising public awareness about rabies disease, first aid measures of animal bites, ensuring compliance with dog vaccination (as dogs are the main reservoir of the disease) and improving access and affordability of the vaccine, to prevent and control the disease.
- Increase the availability and accessibility of the anti-rabies vaccine in different health care units and centers with low cost or even free.
- Develop research about integration of mobile applications and artificial intelligence (AI) in rabies control and surveillance through several innovative approaches.

## Conclusion

The study concluded the effectiveness of the rabies-preventive health program in enhancing mothers' knowledge, attitudes, and practices concerning rabies. A significant improvement in the mothers' overall knowledge about rabies was observed one month after the program's implementation, with highly statistically significant differences. Moreover, there was a notable increase in the total mean attitudinal score following the program, showing statistically significant improvement when compared to pre-program levels. The mothers' practical skills related to rabies prevention also showed marked enhancement one month after the program, with highly statistically significant differences between pre- and post-implementation of program. Additionally, a statistically significant correlation was found between total knowledge scores and total practice scores, both before and one month after the program. The implementation of this program could prevent significant mortality and establish Egypt as a leader in regional disease control efforts, while contributing to the global goal of eliminating dog-mediated rabies deaths by 2030. The research limitations included a relatively small sample size, which potentially compromised the generalizability of findings to the broader population. Furthermore, excluding mothers from rural communities who faced accessibility challenges.

## Data Availability

No datasets were generated or analysed during the current study.
